# Chronic kidney disease as an active driver of digestive tract tumors: mechanistic insights and emerging management strategies

**DOI:** 10.3389/fcell.2026.1797181

**Published:** 2026-03-31

**Authors:** Hanchi Dong, Hongliang Cao, Yirou Gong, Zihan Zhao, Keyan Wang, Xincheng Zhang, Fengchun Zhang

**Affiliations:** 1 Department of Oncology, Suzhou Kowloon Hospital, Shanghai Jiaotong University School of Medicine, Suzhou, Jiangsu, China; 2 China-Japan Union Hospital of Jilin University, Changchun, China; 3 Department of Urology II, The First Hospital of Jilin University, Changchun, China; 4 Department of Oncology, Shanghai Ruijin Hospital, Shanghai Jiaotong University School of Medicine, Shanghai, China

**Keywords:** chronic kidney disease, colorectal cancer, digestive tract tumor, gastric cancer, management, mechanism, microbiome, uremic toxins

## Abstract

Digestive tract tumors (DTT), particularly gastric cancer (GC) and colorectal cancer (CRC), remain among the leading causes of cancer-related morbidity and mortality worldwide. Accumulating epidemiological evidence indicates that patients with chronic kidney disease (CKD) exhibit a significantly increased risk of developing gastrointestinal malignancies and experience worse clinical outcomes. However, the biological mechanisms underlying this association have not been comprehensively synthesized. In this review, we integrate clinical and experimental evidence to delineate how CKD functions as a systemic pro-tumorigenic condition rather than a passive comorbidity. We highlight three interrelated mechanistic axes linking CKD to DTT: (i) persistent systemic inflammation and oxidative stress, (ii) metabolic and endocrine dysregulation driven by uremic toxin accumulation, vitamin D deficiency, and mineral imbalance, and (iii) immune perturbations associated with dialysis modalities and post-transplant immunosuppression. These processes converge to disrupt gastrointestinal barrier integrity, reshape the gut microbiota, impair antitumor immune surveillance, and promote malignant transformation and tumor progression. Importantly, we discuss how CKD-specific interventions, including dialysis strategies, kidney transplantation, dietary management, and modulation of gut microbiota, may further modify gastrointestinal cancer risk. Finally, we propose CKD-oriented preventive and screening strategies for GC and CRC, emphasizing the need for risk stratification based on renal function, proteinuria, and metabolic profiles. By framing CKD as an active driver of gastrointestinal carcinogenesis, this review provides a novel integrative framework that synthesizes interconnected mechanistic pathways and explicitly links them to CKD-specific clinical management strategies, a translational perspective that informs early detection, prevention, and integrated care of DTT in patients with CKD.

## 1 Introduction

Digestive tract tumors (DTT) refer to primary and metastatic malignancies arising in the oral cavity, pharynx, esophagus, stomach, small intestine (duodenum, jejunum, and ileum), and large intestine. Globally, the overall burden of DTT is increasing and shows a shift toward younger onset; both incidence and mortality continue to rise, particularly among males (Schell et al., 2022; Huang et al., 2024; Danpanichkul et al., 2024). This pattern may reflect multiple direct or indirect drivers, including population aging, the growing prevalence of the “three highs” (high blood glucose, hyperlipidemia, and hypertension), and the non-specific and insidious symptoms at early stages (Ahn et al., 2024; Li et al., 2018; Hashimoto et al., 2025). Among these factors, chronic kidney disease (CKD) has emerged as a clinically significant contributor to the development of gastric cancer (GC) and colorectal cancer (CRC). This association underscores the need to clarify CKD-related mechanisms and to investigate why patients with CKD are more susceptible to these cancers, particularly GC and CRC.

CKD refers to a group of kidney disorders of any etiology characterized by structural or functional abnormalities that adversely affect health (Romagnani et al., 2025). According to Kidney Disease: Improving Global Outcomes 2024 (KDIGO 2024), a diagnosis requires kidney abnormalities persisting for at least 3 months, as evidenced by imaging or urine abnormalities, a history of kidney transplantation, or a glomerular filtration rate (GFR) < 60 mL/min/1.73 m^2^ (KDIGO, 2024, 2024). CKD is staged from G1 to G5, and early clinical manifestations are often subtle (Francis et al., 2024). With disease progression, multisystem symptoms emerge. These include anorexia, weight loss, and an increased predisposition to gastrointestinal bleeding. Advanced stages may involve severe uremic complications such as pericarditis, acute pulmonary edema, severe anemia, and uremic encephalopathy (Zoccali et al., 2023; Wang et al., 2025; Rhee et al., 2022; Kalantar-Zadeh et al., 2022; Frąk et al., 2024). Emerging evidence indicates that patients with CKD are particularly susceptible to DTT, which may be driven by inflammatory, metabolic, and treatment-related factors; however, the mechanistic basis remains incompletely defined. While previous articles have individually addressed inflammation, uremic toxins, or microbiota alterations in CKD, a comprehensive synthesis is currently lacking. Such a synthesis would need to integrate these interconnected mechanisms and explicitly translate them into CKD-oriented screening and management strategies. This review synthesizes observational evidence on DTT in CKD, delineates plausible mechanistic pathways, and proposes practical, CKD-specific strategies to preserve kidney and gastrointestinal health and optimize management to improve outcomes.

## 2 Observational evidences suggest a strong association between CKD and DTT

Recent observational studies indicate that patients with renal insufficiency exhibit an increased risk of DTT (Table 1). In a Korean cohort study, Hyung Jung Oh et al. enrolled 35,443 pre-dialysis CKD patients and determined the risk of DTT using the standardized incidence ratio (SIR). Over a 54.9-month follow-up, the risk of DTT in pre-dialysis CKD patients was significantly higher than in the general population (SIR 1.54, 95% CI 1.46–1.62), with respective SIRs for GC and CRC of 1.60 and 1.25 (Oh et al., 2018). Importantly, among CKD patients younger than 40, the incidence of CRC increased markedly compared to their peers in the general population (SIR 4.58) (Oh et al., 2018). While this study provides robust evidence of an association, it is important to acknowledge that such registry-based comparisons may not fully account for the uneven distribution of traditional risk factors. For instance, the higher prevalence of metabolic syndrome components, such as diabetes and obesity, in the general population, and their potential under-documentation in registry data, could confound the observed SIRs, potentially either inflating or masking the true effect of CKD.

**TABLE 1:** Observational studies reveal a close association between CKD and DTT.

Country	First author/Year	Sample size	Population type	Cancer type	Main outcomes	Reference
Korea	Hyung Jung Oh/2018	35,443	Pre-dialysis	GC/CRC	1. CKD patients before dialysis had a significantly higher risk of developing DTT compared to the cohort population (SIR 1.54, 95% CI 1.46–1.62). 2. The probability of developing CRC was significantly increased in CKD patients under the age of 40 (SIR 4.58)	Oh et al. (2018)
Japan	Nanami Kida/2022	18,055	Dialysis	GC/CRC	1. GC and CRC were diagnosed at earlier stages in dialysis patients. 2. Cox proportional hazards models revealed that dialysis patients with CRC or GC had significantly higher mortality compared to their non-dialysis counterparts (both *p* < 0.05)	Kida et al. (2022)
China	Yi-Che Lee/2018	35,928	PD/HD	GC/CRC	1. The risk of GC and CRC significantly increased in patients in the dialysis group. 2. The risk of GC in PD group was approximately twice as high as that in HD group	Lee et al. (2018)
Korea	Jisun Myung/2020	14,382	HD	CRC	CRC was the most common primary site of cancer in both males and females of the dialysis patients	Myung et al. (2020)
Korea	Soon Kil Kwon/2019	48,315	PD/HD	CRC	1. CRC was the most common malignant tumor in dialysis patients. 2. PD had a higher risk of malignant tumors than HD (adjusted HRs: 1.91; 95% CI: 1.59–1.79 vs. 1.69; 95% CI: 1.66–2.2, respectively)	Kwon et al. (2019)
Australia	Eric H Au/2022	1,706	Dialysis	CRC	Among 1706 patients who underwent screening for CKD stages 3–5 based on FIT, 117 cases (6.9%) were found to have advanced CRC.	Au et al. (2022)
Korea	Boyoung Park/2019	10,085	Transplant	GC	1. Kidney transplantation brought a higher DTT risk. 2. GC was more prevalent in male kidney transplant patients	Park et al. (2019)
Germany	Karin Walschburger-Zorn/2013	1,882	Transplant	GC/CRC	1. The probability of DTT in kidney transplant patients was second only to that of urinary system tumors. 2. Both GC and CRC were significantly more prevalent than other types in both males and females	Apel et al. (2013)
Sweden	Britta Krynitz/2013	7,952	Transplant	GC/CRC	1. The cumulative incidence of cancer within 20 years after kidney transplantation was 12%. 2. The SIRs for GC and CRC reached 1.8 and 2.3, respectively	Krynitz et al. (2013)
Japan	Yoshihisa Miyamoto/2023	21,978	-	GC/CRC	Low eGFR was not significantly associated with an increased risk of overall cancers or specific cancers (such as GC and CRC) in CKD patients. This null finding may be explained by several limitations, including inadequate sample size and an eGFR estimation based solely on serum creatinine without information on quantitative albuminuria or serum cystatin C	Miyamoto et al. (2023)

Abbreviation: CKD, chronic kidney disease; DTT, digestive tract tumor; GC, gastric cancer; CRC, colorectal cancer; PD, peritoneal dialysis; HD, hemodialysis; SIR, standardized incidence ratio; FIT, fecal immune test; eGFR, estimated glomerular filtration rate.

A multicenter retrospective cohort study in Japan analyzed 2,161 dialysis patients and 158,964 non-dialysis cancer patients. It found that GC and CRC were diagnosed at earlier stages in the dialysis group. Additionally, cox proportional hazards models revealed that dialysis patients with CRC or GC had significantly higher mortality compared to their non-dialysis counterparts (both *p* < 0.05) (Kida et al., 2022). Additionally, in a retrospective cohort study by Lee et al., 13,473 dialysis patients and 22,455 non-dialysis patients were included. After propensity score matching, the dialysis group demonstrated a significantly higher risk of both GC and CRC. Moreover, patients receiving peritoneal dialysis (PD) presented approximately double the risk of GC compared to those receiving hemodialysis (HD), while no significant difference in CRC risk was observed (Lee et al., 2018). This suggests dialysis modality may influence DTT risk. In South Korea, a cohort study followed 14,382 dialysis patients, among whom 1,124 (7.82%) were diagnosed with cancer during follow-up. Notably, CRC was identified as the most common primary cancer site in both males and females, while stomach cancer ranked third in men and fourth in women (Myung et al., 2020). Additionally, a retrospective analysis of 48,315 dialysis patients and healthy controls similarly identified CRC as the most prevalent malignancy, and the risk of malignancy was observed to be higher in peritoneal dialysis patients than in those undergoing hemodialysis (adjusted HRs: 1.91; 95% CI: 1.59–1.79 vs. 1.69; 95% CI: 1.66–2.2, respectively) (Kwon et al., 2019). Despite adjustments, confounding by indication remains a concern in studies comparing dialysis modalities. Patients are typically selected for PD or HD based on specific clinical profiles, such as age, cardiovascular stability, and diabetic status. These very factors, particularly diabetes and its associated microvascular complications, are also independent risk factors for carcinogenesis. Therefore, the higher GC risk observed in PD patients might be partially attributable to a higher baseline prevalence of diabetes or other unmeasured metabolic disturbances in this group, rather than the dialysis modality itself.

Furthermore, in a prospective cohort study by Eric H. Au et al. involving 1,706 patients with CKD stages 3–5 who underwent fecal immunochemical test (FIT)-based screening, advanced colorectal neoplasia was detected in 117 individuals (6.9%). Subsequent multivariate analysis identified several independent risk factors, including older age (OR, 1.05 per year; 95% CI, 1.03–1.07; *p* < 0.001), male sex (OR, 2.27; 95% CI, 1.45–3.54; *p* < 0.001), azathioprine use (OR, 2.99; 95% CI, 1.40–6.37; *p* = 0.005), and the use of erythropoiesis-stimulating agents (OR, 1.92; 95% CI, 1.22–3.03; *p* = 0.005) (Au et al., 2022). These factors appear to collectively contribute to an elevated risk, suggesting that such patient profiles warrant careful consideration when devising screening protocols. This study is notable for its prospective design and adjustment for multiple variables. However, it also highlights the difficulty of disentangling the effects of CKD from those of its treatments (e.g., immunosuppressants, erythropoiesis-stimulating agents [ESAs]). Furthermore, lifestyle factors notoriously difficult to measure precisely in epidemiological studies, such as smoking status and dietary patterns, were not included in the final multivariate model, representing a potential source of residual confounding.

Moreover, *de novo* malignancies (DNM) are regarded as serious complications following transplantation, with the risk of developing such malignancies in solid organ transplant recipients being 2 to 3 times higher than in the general population (Mazzucotelli et al., 2017; Pradere et al., 2020). In a Korean cohort study comprising 10,085 kidney transplant recipients, Park et al. reported that kidney transplantation was associated with an increased risk of DTT, with GC occurring at a notably higher rate among male recipients (Park et al., 2019). In a retrospective study, Zorn et al. observed that, among kidney transplant patients, the incidence of DTT was second only to that of urological tumors during 9.9 years, and that both GC and CRC were significantly more prevalent than other types in both males and females (Apel et al., 2013). Additionally, a Swedish cohort study analyzing 7,952 CKD patients who underwent kidney transplantation between 1970 and 2008 reported a cumulative cancer incidence of 12% within 20 years after surgery. However, the SIRs for GC and CRC reached 1.8 and 2.3, respectively (Krynitz et al., 2013). Furthermore, mounting evidence indicates that the overall cumulative exposure to immunosuppression remains the key determinant of cancer risk after kidney transplantation, superseding the role of individual drug classes (Vallianou et al., 2025; Sprangers et al., 2018). While the link between immunosuppression and cancer is well-established, interpreting post-transplant cancer risks requires careful consideration of pre-transplant exposures. The elevated SIRs for GC and CRC could reflect not only the oncogenic effects of immunosuppressive drugs but also the legacy of pre-existing uremia, dialysis vintage, and the accumulation of traditional risk factors like smoking, all of which are highly prevalent in this population. The lack of detailed data on pre-transplant lifestyle factors and the duration and severity of prior CKD in many registry studies limits the ability to definitively isolate the contribution of transplantation from the patients' cumulative exposure history. This understanding warrants further investigation into the potential differential effects of specific agents, particularly mTOR inhibitors, on oncogenic outcomes. Collectively, these observational studies suggest that CKD patients exhibit significant gastrointestinal health disparities compared to healthy individuals, and they experience a higher risk of malignancy. Furthermore, CKD-related treatments, including dialysis and kidney transplantation, appear to be associated with the occurrence and progression of DTT. Conversely, evidence from the opposite perspective exists. In a large prospective study, Miyamoto et al. found that low eGFR was not significantly associated with an increased risk of overall cancers or specific cancers (such as GC and CRC) in CKD patients. This null finding may be explained by several limitations, including inadequate sample size and an eGFR estimation based solely on serum creatinine without information on quantitative albuminuria or serum cystatin C (Miyamoto et al., 2023), which underscores the critical need for future studies to incorporate more precise measures of kidney damage, such as proteinuria, alongside comprehensive data on confounders like smoking, obesity, and diabetes, to accurately delineate the independent role of CKD. Meanwhile, there is no research indicating an association between CKD and specific DTT subtypes, and further research is needed.

## 3 Potential mechanisms underlying the multifaceted effects of CKD and DTT

Emerging evidence suggests that the association between CKD and DTT is not driven by a single pathogenic pathway but instead arises from a network of interrelated systemic disturbances. Conceptually, CKD establishes a pro-tumorigenic milieu through three converging mechanistic axes: (i) persistent systemic inflammation and oxidative stress, (ii) metabolic and endocrine dysregulation resulting from uremic toxin accumulation, vitamin D deficiency, and mineral imbalance, and (iii) immune perturbations induced by renal replacement therapies, including dialysis and kidney transplantation. These axes do not operate in isolation; rather, they interact to disrupt gastrointestinal barrier integrity, reshape the gut microbiota, impair antitumor immune surveillance, and promote malignant transformation and tumor progression. In the following sections, we delineate these mechanisms in detail, thereby understanding how CKD actively contributes to the initiation and progression of gastrointestinal malignancies.

### 3.1 CKD-mediated chronic inflammation and oxidative stress elevate the risk of carcinogenesis

CKD is a state of chronic systemic inflammation and oxidative stress, which drives both renal deterioration and increases the risk of GC and CRC. In patients with CKD, hypertension arises from activation of the renin–angiotensin–aldosterone system (RAAS), water and sodium retention, and sympathetic overactivity; sustained hypertension further accelerates renal fibrosis through glomerular hypertension and endothelial injury (De Pascalis et al., 2024; Ku et al., 2019). Within these pathways, angiotensin II (Ang II) and aldosterone play pivotal roles. Excess Ang II and aldosterone activate NADPH oxidase and the NOD-like receptor thermal protein domain-associated protein 3 (NLRP3) inflammasome via the angiotensin II type 1 receptor (AT1R) and the mineralocorticoid receptor (MR), respectively. The resulting activation of NF-κB (nuclear factor kappa-B) and JAK–STAT signaling drives the production of reactive oxygen species (ROS) and pro-inflammatory mediators, including interleukin-6 (IL-6), tumor necrosis factor-αlpha (TNF-α), and interleukin-1 beta (IL-1β), thereby establishing an inflammation–oxidation amplification loop (Zhang X. et al., 2019; Huang et al., 2021; Bruder-Nascimento et al., 2016; Es et al., 2022; Patel et al., 2019). Mechanistically, Ang II promotes assembly of the NLRP3 inflammasome by facilitating NLRP3–ASC (apoptosis - associated speck - like protein containing a CARD) complex formation and upregulating caspase-1 through AT1R and a Ca^2+^-dependent pathway, leading to IL-1β processing and secretion (Zhang X. et al., 2019; Es et al., 2022). By contrast, aldosterone induces vascular injury through interleukin-1 receptor (IL-1R) activation (Bruder-Nascimento et al., 2016). Consistent with these mechanisms, Espitia-Corredor et al. showed that 16-h Ang II exposure in myocardial fibroblasts induces pronounced perinuclear/nuclear colocalization of NLRP3 and ASC, increases caspase-1 activity by approximately 30%, and triggers IL-1β secretion via an AT1R/PLC/IP3R/Ca2+-dependent mechanism (Es et al., 2022). Moreover, Nascimento et al. reported that *IL-1R* ^*−/−*^ and *NLRP3* ^*−/−*^ mice infused with aldosterone for 2 weeks are protected from aldosterone-induced vascular dysfunction, evidenced by suppression of VCAM-1 and ICAM-1 expression, thereby highlighting essential roles for IL-1β and NLRP3 in aldosterone-induced vascular damage and underscoring the contribution of hypertension and aldosterone to inflammasome activation (Bruder-Nascimento et al., 2016). However, extrapolating these findings from animal models to humans requires caution.

**FIGURE 1:**
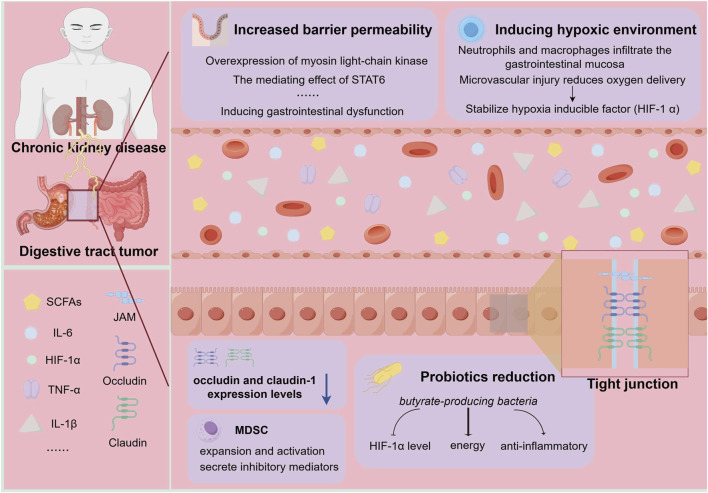
Inflammatory mediators disrupt gastrointestinal homeostasis. Inflammatory factors increase the permeability of the gastrointestinal barrier through mechanisms that involve MLCK overexpression and STAT6-mediated effects, among others. Subsequently, MLCK-dependent phosphorylation of the regulatory MLC is responsible for the activation of myosin II and the contraction of the peri-junction actin ring. STAT6 activates downstream target MLCK1, leading to disruption of tight junction structures in epithelial cells. In addition, infiltration of neutrophils, macrophages, and other cells into the gastrointestinal mucosa markedly increases oxygen consumption, while microvascular damage reduces oxygen delivery; together, these changes lead to tissue hypoxia, which further stabilizes HIF-1α. Moreover, inflammatory factors regulate the expression of tight junction proteins, with significant reductions in occludin and claudin-1 levels. At the same time, HIF-1α induces the expansion and activation of MDSCs, which subsequently secrete inhibitory mediators, creating an immunosuppressive microenvironment and increasing the likelihood of tumorigenesis. Furthermore, the inflammatory–hypoxic environment in the gut also affects microbiota homeostasis, characterized by a decrease in butyrate-producing bacteria. This prevents the effective downregulation of HIF-1α and simultaneously weakens the energy supply and anti-inflammatory capacity of intestinal epithelial cells, thereby exacerbating inflammation and markedly elevating the risk of DTT. MLCK, myosin light-chain kinase; STAT6, signal transducer and activator of transcription 6; MLC, myosin light-chain; MLCK1, myosin light-chain kinase 1; MDSC, myeloid-derived suppressor cells; HIF-1α, hypoxia-inducible factor-1α; DTT, digestive tract tumor; SCFAs, short-chain fatty acids; IL-6, interleukin-6; TNF-α, tumor necrosis factor-alpha; IL-1β, interleukin-1 beta (By Figdraw).

Moreover, evidence indicates that inflammatory mediators enter the gastrointestinal mucosa via the circulation, increasing local microvascular permeability and driving structural remodeling, thereby exacerbating gastrointestinal barrier dysfunction (Figure 1) (Britzen-Laurent et al., 2023; Li et al., 2025). Convergent data from cellular, animal, and human tissue studies demonstrate that inflammatory stimuli heighten barrier permeability through mechanisms that include overexpression of MLCK (myosin light-chain kinase) and mediation by *STAT6* (*signal transducer and activator of transcription 6*) (Meyer et al., 2023; Jin and Blikslager, 2020; Lin et al., 2019). Concurrently, infiltration of inflammatory cells (e.g., neutrophils and macrophages) into the gastrointestinal mucosa markedly elevates oxygen consumption, while microvascular injury diminishes oxygen delivery; together, these processes induce tissue hypoxia, which stabilizes hypoxia-inducible factor-1α (HIF-1α) and establishes a positive feedback loop (Lun et al., 2023). In a rat model of sepsis, Lei et al. reported significantly higher HIF-1α expression in septic animals versus controls (*p* < 0.05), along with pronounced hyperplasia of the intestinal lamina propria and extensive protrusion and sloughing of villus epithelium. They further observed significantly reduced expression of the tight junction proteins ZO-1, occludin, and claudin-1, underscoring the pivotal role of HIF-1α in barrier impairment (Lei et al., 2022). Under chronic inflammatory hypoxia, HIF-1α also promotes the expansion and activation of myeloid-derived suppressor cells (MDSCs) (Köstlin-Gille et al., 2019). By secreting inhibitory mediators (e.g., interleukin-10 [IL-10], inducible nitric oxide synthase [iNOS], and vascular endothelial growth factor [VEGF]) and depleting local arginine, MDSCs suppress T-cell function and create an immunosuppressive microenvironment (Tran et al., 2020; Daniel et al., 2019), thereby undermining immune surveillance and increasing the risk of tissue carcinogenesis. Moreover, a hypoxic and inflammatory intestinal environment reduces butyrate-producing bacteria, which both impairs effective downregulation of HIF-1α and diminishes the energy supply and anti-inflammatory capacity of intestinal epithelial cells. These changes exacerbate inflammation and markedly increase the risk of DTT (He et al., 2022; Dengler et al., 2021). In *in vitro* studies, butyrate lowers HIF-1α levels, likely preventing its accumulation by inhibiting lactate dehydrogenase A (LDHA) activity (Zhao et al., 2024; Nishioku et al., 2025; Xiong et al., 2025; Yan et al., 2024). Furthermore, chronic inflammation critically modulates cancer stemness to drive tumor initiation and malignant progression (Wang T. et al., 2019). Specifically, inflammatory mediators induce deacetylation of FOS-related antigen 1 (FRA1), imparting stem-like properties to CRC cells—enhancing self-renewal, differentiation, and therapy resistance (Wang T. et al., 2019)—thereby directly promoting CRC progression.

Patients with CKD exhibit a marked imbalance in oxidative stress, characterized by the accumulation of ROS and diminished antioxidant capacity; this state persists across all stages of CKD (Roumeliotis et al., 2024; Tsinari et al., 2025). The gastrointestinal tract is a primary site of ROS generation. Excess ROS perturbs cellular metabolism by inducing DNA damage, lipid peroxidation, and protein oxidation. It can directly attack DNA, giving rise to carcinogenic mutations (Ohno et al., 2024; Li J. et al., 2024). At the tissue level, Scalise et al. showed that DNA damage may induce *TP53* mutations, thereby initiating CRC (Scalise et al., 2016). Moreover, ROS may compromise base excision repair glycosylases (hOGG1, MUTYH), leading to mutation accumulation and promoting the progression of DTT (Vodicka et al., 2020). In gastrointestinal cells, lipid peroxidation damages mitochondrial membranes, lowering mitochondrial membrane potential and impairing electron transport chain function, thereby reducing energy supply (Zheng et al., 2025). Furthermore, growing evidence highlights the central role of cardiolipin in mitochondrial regulation, including the assembly of complexes III and IV and the maintenance of cristae architecture. However, cardiolipin’s conical geometry and specific localization to negatively curved membranes render it highly susceptible to oxidative modification, resulting in mitochondrial dysfunction (El-Hafidi et al., 2020). In addition, ROS also induces carbonylation of protein side chains, leading to enzyme inactivation and structural protein denaturation. Consequently, antioxidant enzyme activities in the gastrointestinal mucosa (e.g., superoxide dismutase and catalase) are suppressed, promoting oxidant accumulation and exacerbating tissue injury (Vona et al., 2021; Choudhary et al., 2022). Inflammation and oxidative stress form a vicious cycle: CKD-associated inflammation amplifies oxidative stress, which in turn enhances the release of inflammatory mediators. This cycle not only damages the kidneys but also accelerates the initiation and progression of DTT.

### 3.2 CKD-related metabolic alterations promote the development of DTT

**FIGURE 2:**
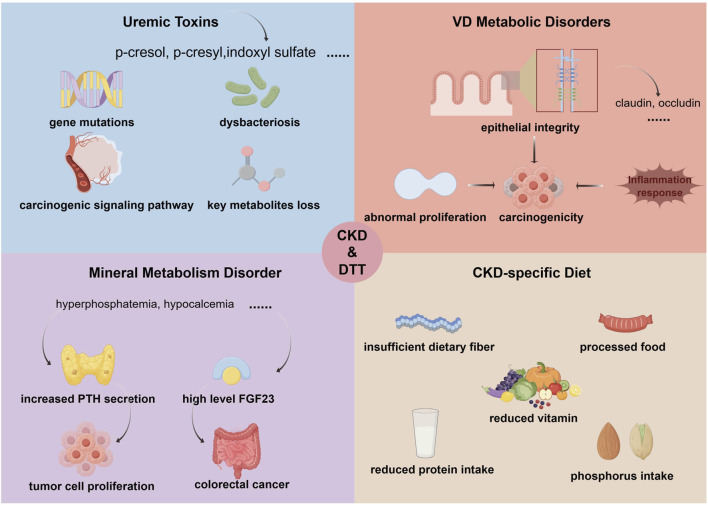
Metabolic alterations in CKD drive the development of DTT. CKD–related metabolic alterations, including the accumulation of uremic toxins, disrupted VD metabolism, mineral metabolism disorders, and CKD-specific dietary practices, collectively contribute, to varying degrees, to the initiation and progression of DTT. Intestinal uremic toxins, notably p-cresol, p-cresyl sulfate, and indoxyl sulfate, reshape the gut microenvironment, promote dysbiosis, weaken anti-tumor defenses, and may directly compromise genomic stability, while simultaneously depleting protective metabolites such as SCFAs. Perturbations in VD metabolism further undermine gastrointestinal homeostasis by impairing epithelial integrity, dysregulating anti-inflammatory immune responses, and permitting aberrant cellular proliferation. In parallel, mineral disorders characterized by hyperphosphatemia, hypocalcemia, and elevated PTH indirectly accelerate DTT progression, and increased FGF23 has been implicated as a meaningful marker for CRC diagnosis and screening. Additionally, dietary regimens common in CKD care (low potassium, low phosphorus, and protein restriction) may influence DTT risk via altered nutrient intake and absorption; notably, the relationship between phosphorus intake and DTT risk remains to be clarified and warrants further investigation. Collectively, these CKD-associated metabolic disturbances appear to partially drive the development of DTT. CKD, chronic kidney disease; DTT, digestive tract tumor; CRC, colorectal cancer; VD, vitamin D; SCFAs, short-chain fatty acids; PTH, parathyroid hormone; FGF23, fibroblast growth factor 23 (By Figdraw).

Metabolic factors associated with CKD, including uremic toxin accumulation, disordered vitamin D (VD) metabolism, mineral metabolism disturbances, and CKD-specific dietary patterns, collectively contribute, to varying degrees, to the initiation and progression of DTT (Figure 2). Declining renal function in CKD frequently leads to the buildup of uremic toxins. These intestinal uremic toxins alter the gut microenvironment and promote dysbiosis in CKD patients. Characteristically, dysbiosis involves an expansion of aerobic bacterial strains, which drives the production of three major uremic toxins: p-cresol, p-cresyl sulfate, and indoxyl sulfate (Ramezani and Raj, 2014; Di et al., 2023). Notably, Ichisaka et al. linked CKD progression to worsening CRC. Treating HCT-116 CRC cells with indoxyl sulfate for 24 h elevated c-Myc protein levels and promoted cell proliferation via activation of the Akt/β-catenin/c-Myc signaling pathway (Ichisaka et al., 2025). In addition, experiments have shown that indoxyl sulfate significantly enhances the proliferation ability of CRC cells at concentrations exceeding 62.5 µ M, which is inhibited by the aryl hydrocarbon receptor (AhR) antagonist CH223191, demonstrating the direct role of the AhR/c-Myc signaling pathway in proliferation (Ichisaka et al., 2025; Ichisaka et al., 2024). These findings indicate that uremic toxins play a critical mediating role in the CKD–CRC axis and highlight them as potential therapeutic targets in CKD. Furthermore, dysbiosis induced by CKD-related uremic toxin accumulation undermines anti-tumor defenses and can directly compromise genomic stability through multiple mechanisms, including depletion of protective metabolites (e.g., short-chain fatty acids, SCFAs), induction of oxidative DNA damage, mutations in key genes (e.g., *APC* or *KRAS*), and activation of oncogenic signaling pathways (e.g., NF-κB) (Kuru-Yaşar and Üstün-Aytekin, 2024; García et al., 2024; Li Y. et al., 2024), thereby promoting DTT.

In addition, VD plays a critical role in maintaining intestinal epithelial integrity, modulating immune responses (predominantly anti-inflammatory), and suppressing aberrant cellular proliferation. Patients with CKD frequently present with VD deficiency (<20 ng/mL) or insufficiency (20–29 ng/mL) (Jean et al., 2017). Impaired renal function diminishes the conversion of 25-hydroxyvitamin D to its active metabolite, 1,25-dihydroxyvitamin D, resulting in reduced circulating levels of active VD (Tang et al., 2025). Active VD binds the vitamin D receptor (VDR) in intestinal epithelial cells to regulate the expression of tight junction proteins, such as occludin and claudins, thereby preserving mucosal barrier integrity and permeability (Assa et al., 2015; Yang et al., 2024). In states of deficiency, this barrier is compromised; translocation of bacteria and endotoxins across the mucosa exacerbates chronic inflammation and elevates cancer risk. In a cross-sectional study, Kwak et al. reported that sufficient VD levels were associated with a reduced risk of *Helicobacter pylori* infection, a major etiological factor for GC (Kwak and Paik, 2020). Furthermore, using CRC mouse models and clinical serum samples, Zhou et al. showed that VD supplementation improved disease indices and inhibited CRC initiation and progression. Their work also highlighted VD’s key regulatory role in maintaining colonic barrier integrity via gut probiotics and *A. muciniphila* (Zhou et al., 2020). Notably, some researchers found that VDR-deficient mouse models exhibited reduced colonic expression of the barrier protein claudin-5, along with significantly increased levels of the proliferation marker PCNA (proliferating cell nuclear antigen), compared with controls (Zhang et al., 2022; Sun and Zhang, 2022). In addition, the VD/VDR signaling axis also helps regulate gut microbial homeostasis and promotes colonization by probiotics such as *Bifidobacterium* and *Lactobacillus* (Sharma et al., 2023). Consequently, CKD-associated reductions in probiotic diversity, together with a loss of SCFA-producing taxa (Zeb et al., 2025), further impair the epithelial barrier, amplify inflammation, and foster a tumor-promoting microenvironment.

Moreover, disturbances in mineral metabolism, specifically hyperphosphatemia, hypocalcemia, and elevated parathyroid hormone (PTH), are important drivers of DTT in patients with CKD. Previous studies have shown that elevated phosphate stimulates secretion of fibroblast growth factor 23 (FGF23) (Vogt et al., 2019; Zhou W. et al., 2023; Hamid et al., 2024). High circulating FGF23 has been implicated, particularly in CRC, as a clinically relevant factor for diagnosis and screening, and may promote carcinogenesis by enhancing inflammation and angiogenesis or by directly stimulating epithelial cell proliferation (Wang et al., 2014). Additionally, phosphate excess and calcium deficiency also trigger PTH release (Ketteler et al., 2025). Hyperparathyroidism states may increase cancer risk indirectly by modulating calcium-dependent signaling, including the Wnt/β-catenin pathway, or by promoting cellular proliferation (Wang W. et al., 2019; Takeda et al., 2017; Zhang K. et al., 2019). Notably, Jacklyn N. Hellwege and colleagues evaluated the joint effects of VD deficiency and PTH responsiveness on CRC risk in a prospective cohort. Under VD deficiency, PTH hypo-responsivity was associated with a 2.56-fold increase in CRC risk, whereas PTH hyperresponsiveness conferred only a non-significant 1.65-fold increase (Hellwege et al., 2021). These findings suggest that the combined VD–PTH status may represent a potential biomarker.

Distal to renal pathology, dietary management strategies for CKD, including low-potassium, low-phosphorus, and protein-restricted regimens, also appear to be associated with DTT. Clinicians commonly advise patients to limit potassium-rich fruits and vegetables (e.g., bananas, oranges, tomatoes, spinach, potatoes), which are rich in dietary fiber and antioxidants such as vitamin C and carotenoids. Such restrictions may reduce fiber intake and decrease consumption of antioxidant vitamins and phytochemicals. Evidence suggests that inadequate fiber intake is a significant risk factor for DTT, particularly CRC (Gianfredi et al., 2018; Cifuentes and O'Keefe, 2024). By lowering the inflammatory potential of the diet, as reflected in reduced E-DII (Energy-adjusted Dietary Inflammatory Index) scores, fiber may attenuate CRC-related Wnt signaling activity and thereby inhibit CRC progression (Malcomson et al., 2021). Besides, antioxidant vitamins (A, C, and E) and phytochemicals (e.g., polyphenols and carotenoids) further contribute by neutralizing free radicals and enhancing DNA repair (Fenech et al., 2023). Multiple studies reported that vitamins and polyphenols significantly lowered the oxidative stress biomarker 8-OHdG (El et al., 2023; Ilari et al., 2025; Tratenšek et al., 2024), which may indirectly reduce cancer risk. High-phosphorus foods such as dairy products and nuts are likewise often limited. However, a recent cross-sectional study observed an inverse association between phosphorus intake and CRC risk (Qin et al., 2025). In contrast, Ye et al. demonstrated in a CKD rat model that high-phosphorus feeding induces detrimental alterations in the gut microbiota and adversely affects long-term health outcomes (Ye et al., 2021). Taken together, the relationship between dietary phosphorus and DTT risk remains inconsistent across populations and disease stages, particularly in CKD; high-quality CKD-specific prospective data are needed. Protein intake is also commonly restricted to reduce renal workload and the generation of uremic toxins. To meet energy and essential amino acid requirements under such constraints, some patients may rely more heavily on animal protein, particularly red and processed meats. Yet high consumption of these foods is a well-established risk factor for CRC (Ungvari et al., 2025). Quantitatively, Knuppel et al. reported that each 50 g increment in red meat intake confers an approximately 36% higher CRC risk (HR 1.36, 95% CI: 1.13–1.64) (Knuppel et al., 2020). Collectively, CKD-related metabolic and dietary changes may promote DTT progression through multiple pathways, while simultaneously revealing potential opportunities to manage DTT through intervention in CKD. Notably, most mechanistic data derive from preclinical models; prospective human studies integrating inflammatory and metabolic biomarkers are needed to validate causality and therapeutic targets in CKD-associated GC/CRC.

### 3.3 Dialysis and kidney transplantation further increase the risk

Dialysis is a non-physiologic intervention in which blood contacts bioincompatible membranes and dialysate components, repeatedly activating the immune system and triggering the release of inflammatory cytokines, including IL-1β, IL-6, and TNF-α (Chang et al., 2020). Dialysis may also induce an acquired immunodeficiency, reflected by abnormal monocyte activation driven by dialysis membranes and dialysate (Carracedo et al., 2002). Despite this, cellulose membranes, particularly cellulose acetate derivatives, remain widely used in clinical practice because of their favorable biocompatibility and distinctive advantages for heparin-free dialysis (Puerta et al., 2025; Ragab et al., 2023). Evidence indicated that monocyte exposure to cellulose dialysis membranes promoted robust IL-1β and TNF-α release *in vivo* and *in vitro*, fostering persistent low-grade inflammation. Meanwhile, repeated activation yields a CD14^+^CD32^+^ senescent phenotype and marked telomere shortening in monocytes; during dialysis, complement activation and trace bacterial endotoxin residues further exacerbate monocyte dysfunction (Carracedo et al., 2002; Paul et al., 2022). Collectively, these alterations blunt acute stress immune responses while enhancing the release of pro-tumorigenic factors, thereby increasing cancer risk. Notably, in polysulfone-based dialysis sessions, Vassilios Liakopoulos and colleagues reported a skewing of monocyte subsets in HD patients toward pro-inflammatory CD16^+^ Mo2 and Mo3 populations. They also observed significantly reduced CX3CR1 expression (receptors that promote monocyte migration) in Mo2/Mo3 subsets, specifically to approximately 0.7-fold of the level in healthy controls, with further downregulation post-dialysis. Additionally, leukocyte adhesive capacity was diminished after dialysis compared with pre-dialysis. These findings, however, derive from a relatively small cohort (n = 15) and warrant confirmation in larger studies (Liakopoulos et al., 2018).

Post-transplant immunosuppression is widely recognized to facilitate the development of multiple cancers. Immunosuppressive agents diminish T-cell and natural killer (NK) cell activity, impairing immune surveillance; for instance, this can predispose to Epstein–Barr virus (EBV) infection and subsequent lymphoepithelioma-like gastric carcinoma (Hirabayashi et al., 2023). Immunosuppressants may also exert direct mutagenic effects. Using comet and micronucleus assays, Cilião et al. evaluated DNA instability in blood lymphocytes from 76 kidney transplant recipients and, relative to 17 healthy controls, found that long-term tacrolimus exposure was associated with increased DNA damage and induced genetic instability (Lizotti Cilião et al., 2016). These changes directly promote malignant tumorigenesis at the genetic level, including DTT.

### 3.4 Evidence grading and causal strength

Throughout this review, we have drawn upon a diverse range of studies, including epidemiological observations, clinical cohort analyses, and mechanistic experiments. To enhance interpretability and avoid overinterpretation of associative findings as causal, it is useful to stratify the evidence according to study design and consistency.

Observational epidemiological evidence (Section 2) primarily derives from retrospective and prospective cohort studies. These studies consistently demonstrate an increased risk of GC and CRC in patients with CKD, particularly those on dialysis or after kidney transplantation. However, these designs can only establish association, not causation, due to potential residual confounding (e.g., diabetes, obesity, smoking) and the inability to fully account for competing risks. The study by Miyamoto et al. (Miyamoto et al., 2023) exemplifies null findings that highlight the need for more precise exposure assessment.

Mechanistic evidence (Sections 3.1–3.3) includes *in vitro* cellular experiments, animal models, and *ex vivo* human tissue analyses. These studies provide plausible biological pathways, such as inflammation, oxidative stress, uremic toxin signaling, and immune modulation, that link CKD to gastrointestinal carcinogenesis. While these mechanistic data support causal inference, they are largely derived from preclinical models and require validation in human cohorts with longitudinal designs and biomarker integration.

Evidence consistency and strength: The association between CKD and DTT is supported by multiple large-scale cohort studies across different populations (Korea, Japan, China, Sweden, etc*.*), with particularly elevated risks in younger dialysis patients and post-transplant recipients. The consistency of these findings across diverse settings lends weight to the existence of a true relationship. Nevertheless, the evidence for specific mechanistic pathways remains heterogeneous, and causal mediation has not been definitively proven in humans.

Conclusion on causality: At present, the accumulated evidence supports that CKD is independently associated with an increased risk of DTT, and that multiple interconnected mechanisms are likely involved. However, definitive causal inference awaits future prospective studies that integrate detailed renal phenotyping (e.g., proteinuria, eGFR trajectory), serial biomarker measurements, and careful adjustment for confounders. Readers should interpret the current evidence as indicating strong associations and plausible mechanisms, rather than proven causality.

## 4 Intervention measures for CKD treatment to better manage DTT

**FIGURE 3:**
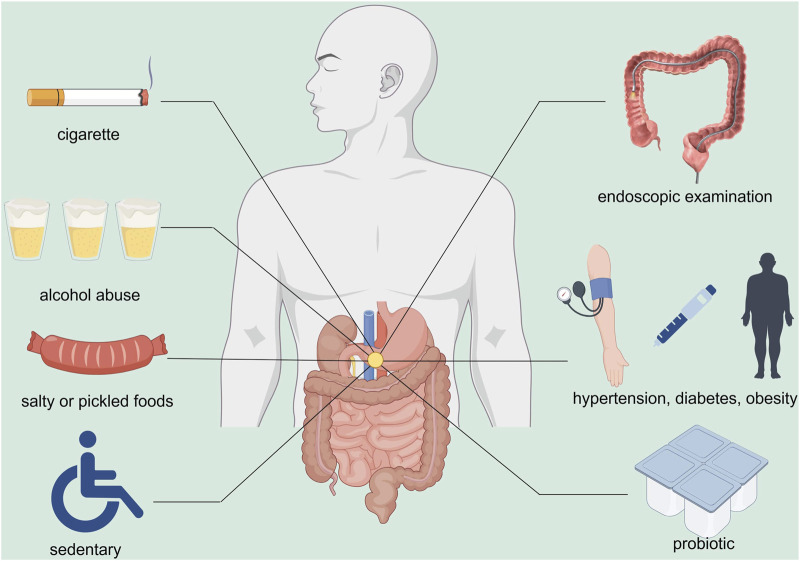
Potential strategies for the management of CKD and DTT. Current evidence suggests that intervention in CKD is regarded as an effective approach to DTT management. Key risk factors for DTT, including smoking, alcohol abuse, consumption of high-salt and preserved foods, and physical inactivity, are believed to adversely impact gastrointestinal health via multiple pathways, thereby promoting tumorigenesis. Probiotic supplementation is recognized as an effective measure, as the maintenance of gut microbiota homeostasis is considered beneficial for gastrointestinal health. Strict control of blood glucose, blood pressure, and BMI, together with regular screenings, is considered essential for the effective management of DTT. DTT, Digestive Tract Tumor; CKD, Chronic Kidney Disease; BMI, Body Mass Index (By Figdraw).

Although kidney injury caused by CKD is irreversible, appropriate interventions are considered effective for treating CKD to improve the management of DTT. In this section, interventions such as lifestyle modification and modulation of gut homeostasis are discussed as approaches to treat CKD and thereby manage DTT (Figure 3).

### 4.1 Stopping bad habits

Unhealthy lifestyle behaviors, such as including smoking, alcohol abuse, consumption of high-salt preserved foods, and physical inactivity, adversely affect renal and gastrointestinal health. Chronic exposure to cigarette smoke, whether through active smoking or secondhand smoke, is a well-established independent risk factor for CKD. Relative to individuals with ≥30 pack-years, those with <15 pack-years have a lower risk of CKD progression (Lee S. et al., 2021). In addition, smoking elevates the risks of vascular disease and cancer-related mortality among patients with CKD, with relative risks (RR) of 1.35 and 2.32, respectively (Staplin et al., 2016). Evidence indicates that nicotine contributes to renal injury and accelerates CKD progression through modulation of the α7 nicotinic acetylcholine receptor (α7nAChR), the NOD-like receptor thermal protein domain-associated protein 6 (NLRP6) inflammasome, endoplasmic reticulum stress, and autophagy (Zheng et al., 2020). Moreover, Maeve T. Morris and colleagues quantified the combined risk: smokers infected with *Helicobacter pylori* had a 2- to 11-fold higher risk of gastric cancer, and cigarette smoke may exacerbate gastric epithelial DNA damage and hasten *H. pylori*–induced dysplasia (Morris et al., 2025). Consequently, smoking cessation is an effective intervention to mitigate these risks.

While alcohol consumption is conventionally recognized as a risk factor, accumulating evidence suggests that moderate intake is associated with a lower risk of CKD. A Korean cohort study demonstrated that individuals consuming 10–30 g/day of alcohol experienced a slower decline in estimated glomerular filtration rate (eGFR) over 12 years compared with non-drinkers (Lee YJ. et al., 2021). Consistently, a meta-analysis reported that light alcohol consumption (<26 g/day) is associated with a 12%–24% reduction in the risk of chronic kidney injury (Li et al., 2019). Conversely, alcohol abuse can aggravate renal injury through mitochondrial dysfunction, oxidative stress, and inflammation, with indirect effects mediated by altered intestinal permeability and the microbiome, hepatic injury, and cardiac dysfunction (Yokus et al., 2025). These alterations also disrupt gastrointestinal homeostasis, increasing susceptibility to DTT (mechanisms previously described). Furthermore, in a nationwide analysis of patients with gastric ulcers, Maksat Idris and colleagues found that individuals with alcohol abuse had the highest rates of perforation or bleeding among all cases. The research also indicated that alcohol abuse was likewise a significant risk factor for gastric ulcers and related pathologies and may elevate the risk of upper gastrointestinal malignancy (Idris et al., 2022).

With rising living standards, consumption of high-salt preserved foods (e.g., pickled vegetables, salted meats) and processed meats (e.g., bacon, sausages) has increased. High salt intake directly impairs renal function by elevating blood pressure and glomerular filtration load. Processed meat products account for roughly 20% of daily sodium intake and constitute important risk factors for CKD (Chen et al., 2023; Wang et al., 2023). In a synthesis of evidence on total red and processed meat consumption, Farvid et al. reported a significant association between red meat intake and CRC risk (RR = 1.10; 95% CI: 1.03–1.17). Processed meat intake was linked to an approximately 18% higher risk of CRC. Consistent with these findings, the study also showed that total consumption of red and processed meats was significantly associated with increased CRC risk (RR = 1.17; 95% CI: 1.08–1.26) (Farvid et al., 2021). A separate meta-analysis found that high salt intake was significantly associated with increased GC risk (RR = 1.25; 95% CI: 1.10–1.41; *P* = 0.001), and that high consumption of preserved foods likewise elevated GC risk (RR = 1.28; 95% CI: 1.05–1.57) (Wu et al., 2021). Notably, high salt intake has also been linked to classic precancerous lesions, including erosive esophagitis, severe atrophy, and intestinal metaplasia (Khasag et al., 2018). Mechanistically, high salt intake compromises gastric mucosal integrity, promotes *H. pylori* colonization, and thereby facilitates GC development (Balendra et al., 2023). Using RNA sequencing, John T. Loh and colleagues compared *H. pylori* grown in media with varying sodium chloride concentrations. They showed that high-salt conditions may modulate bacterial gene expression (e.g., *sabA*, *hopQ*), enhancing adhesion and colonization (Loh et al., 2018). In addition, high-salt and preserved foods contain substantial nitrite, which can be converted to carcinogenic nitrosamines, thereby directly increasing cancer risk (Deveci and Tek, 2024).

Furthermore, sedentary behavior contributes to the progression of CKD and DTT. A Chinese cohort study showed that greater sedentary time was significantly associated with renal function decline, particularly among older adults, independent of other risk factors (e.g., age, hypertension, cancer history) (Yu et al., 2023). Moreover, a comprehensive analysis reported that individuals who were sedentary and performed no physical activity had a significantly higher risk of CRC than their active counterparts (OR 1.49; 95% CI 1.02–2.16) (An and Park, 2022). Mechanistically, sedentary behavior may elevate cancer risk by disrupting sex hormone metabolism, impairing glucose uptake efficiency, and inducing adipokine dysregulation (Friedenreich et al., 2021; Jochem et al., 2019). Consequently, adopting healthy lifestyle practices, including smoking cessation, moderate alcohol consumption, reduced intake of processed foods, and regular moderate exercise, is critical for maintaining renal and gastrointestinal health.

### 4.2 Potential adjustment measures

In CKD, strategies that modulate the gut microbiota, reduce uremic toxin burden, and regulate inflammation and metabolism can reinforce renoprotection and improve management of DTT. Probiotics, prebiotics, and synbiotics play a key role in reshaping the gut microbiota to support kidney function. Evidence indicates that probiotics and synbiotics restore intestinal barrier integrity, increase SCFAs production, and lower uremic toxins such as indoxyl sulfate and p-cresyl sulfate, thereby reducing renal load and slowing CKD progression (Ribeiro et al., 2024). A meta-analysis also suggests that probiotics, prebiotics, and synbiotics may help prevent CRC and precancerous lesions (Kan et al., 2024). In addition, AST-120, an oral spherical carbon adsorbent, is another effective intervention. It significantly reduces the accumulation of protein-bound uremic toxins, including indoxyl sulfate and p-cresyl sulfate (Yamaguchi et al., 2017; Asai et al., 2019), and has shown renoprotective effects in animal models, consistent with decreased toxin levels (Lin et al., 2020). Growing interest in the gut microbiota underscores intestinal microecology modulation as a promising strategy for CKD and DTT management. Moreover, ferric citrate has demonstrated additional benefits. In *Col4α3* knockout mice (a CKD model), it lowered serum phosphate and markedly reduced circulating FGF23, while also diminishing systemic inflammation and improving renal function (Hanudel et al., 2022). In addition, a randomized controlled trial further reported that ferric citrate coordination complex favorably alters biochemical parameters in CKD patients with an acceptable safety profile (Block et al., 2019). As noted in Section 3.1, IL-1β–mediated inflammation is pivotal. In an animal study, soybean-derived soybean phospholipids and soy protein isolate suppressed IL-1β gene expression, highlighting their potential to attenuate chronic inflammation and slow CKD progression (Ohta et al., 2022). Taken together, these interventions provide renal protection while more effectively managing the progression of DTT.

### 4.3 Try to avoid diabetes, hypertension, and obesity

Diabetes is a common risk factor for CKD and DTT, and CKD is recognized as a frequent complication of type 2 diabetes (T2DM) (Rossing et al., 2023). Poor glycemic control is directly associated with a higher risk of CKD in individuals with diabetes. In a Chinese cohort study, participants with uncontrolled hyperglycemia have greater risks of CKD, diabetic kidney disease (DKD), and glomerulonephritis than those with well-controlled blood glucose (Wang et al., 2024). Mechanistically, chronic hyperglycemia accelerates CKD progression by increasing the formation of advanced glycation end products (AGEs), activating protein kinase C (PKC) and JAK signaling, and inducing oxidative stress, which directly injures glomerular endothelial cells, podocytes, and renal tubular epithelial cells (Shetty et al., 2025). In parallel, diabetes is increasingly viewed as a systemic inflammatory condition, and this proinflammatory milieu may foster carcinogenesis by remodeling the tumor microenvironment. Hyperglycemia not only stimulates extracellular matrix (ECM) synthesis but also drives epithelial and endothelial cells toward fibroblast-like phenotypes, activating fibrogenic pathways and indirectly promoting tumorigenesis (Tuleta et al., 2021). Moreover, hyperglycemia further impairs antitumor immunity: it downregulates major histocompatibility complex (MHC) class II molecules (HLA-DR) and costimulatory molecules (CD86), thereby diminishing dendritic cell antigen presentation and CD4^+^ T-cell activation (Monroy-Mérida et al., 2021). Evidence also indicates that high glucose suppresses CD8^+^ T-cell effector functions (Hulme et al., 2024), increases the proportion of regulatory T cells (Tregs) (Pitmon et al., 2023), and reduces natural killer (NK) cell activity (Duan et al., 2019). Collectively, these changes compromise immune surveillance and heighten the risk of malignant transformation in gastrointestinal epithelial cells. Moreover, Chatterjee et al. reported that hyperglycemic conditions induce MMP-9 transcriptional activation, enhancing the invasiveness of gastric adenocarcinoma cells and directly promoting the development of GC (Chatterjee et al., 2024). Accordingly, early and effective glycemic control is critical for mitigating the progression of CKD and DTT.

Chronic hypertension contributes substantially to kidney injury worldwide, causing persistent structural and functional damage and representing a major risk factor for CKD and end-stage renal disease (ESRD). Sustained elevations in glomerular perfusion pressure induce a state of glomerular hypertension and hyperfiltration, which damages capillary walls and endothelial cells and ultimately progresses to glomerulosclerosis with declining filtration capacity (Nagata and Hishida, 2024). At the cellular level, Uruski et al. reported that hypertension promotes tumor cell proliferation, migration, and invasion (Uruski et al., 2024). Given that hypertension-induced disturbances in systemic homeostasis were discussed previously, we do not elaborate further here.

Obesity is an independent risk factor for CKD (Avgoustou et al., 2025) and is closely associated with DTT. It accelerates CKD progression through multiple pathways, including overactivation of the RAAS (Rüster and Wolf, 2013), proinflammatory macrophage phenotypic shifts (Vallianou et al., 2021), and insulin resistance–induced podocyte injury and glomerulosclerosis (Rogacka, 2021). When obesity coexists with CKD, DTT progression is further accelerated. Concurrently, gut microbiome diversity and compositional integrity decline, increasing the production of trimethylamine (TMA) and branched-chain fatty acids (BCFAs) while reducing SCFAs generation (Kounatidis et al., 2024). These alterations disrupt lipid metabolism and amplify inflammatory signaling. The crosstalk among obesity, CKD, and gut dysbiosis is frequently mediated by “leaky gut”, a term referring to dysregulated tight junctions in intestinal epithelial cells, leading to elevated serum lipopolysaccharide (LPS) levels and systemic inflammation (Kounatidis et al., 2024), which indirectly drives DTT progression. Collectively, these interactions create a self-perpetuating cycle that exacerbates DTT pathogenesis. Accordingly, effective body mass index (BMI) management benefits both CKD and DTT. More broadly, rigorous control of blood glucose, blood pressure, and BMI is essential for mitigating the onset and progression of CKD and DTT.

### 4.4 Appropriate screening

The elevated risks of GC and CRC among patients with CKD highlight the clinical importance of early screening. Timely, effective screening can improve outcomes. A comprehensive evaluation should include renal function indices and endoscopic assessment. Regular, detailed monitoring is warranted to detect disease progression and enable prompt adjustments to treatment plans. Accumulating evidence links CKD-related proteinuria dynamics to CRC risk. In a retrospective cohort with a median follow-up of 9.19 years, Soo Young Oh and colleagues reported that persistent proteinuria was associated with a higher risk of CRC compared with no proteinuria (adjusted hazard ratio [aHR], 1.27; 95% CI, 1.13–1.42). The severity of proteinuria was also positively correlated with CRC risk (Oh et al., 2025). These findings provide observational evidence supporting routine surveillance of proteinuria as a potential risk marker; however, prospective studies are needed to validate whether proteinuria-based risk stratification improves clinical outcomes. Furthermore, a Mendelian randomization analysis showed that, after adjustment for BMI and smoking initiation, each 10% decrease in eGFR corresponded to odds ratios of 0.93 (95% CI, 0.65–1.33) for GC and 1.05 (95% CI, 0.90–1.23) for CRC (Tang et al., 2022). Although these associations were not statistically significant, the findings do not preclude a potential role for eGFR in risk assessment, particularly when combined with other clinical parameters. Nevertheless, the predictive value of eGFR for DTT risk requires further investigation in well-designed prospective cohorts.

Moreover, endoscopy remains the most direct and precise modality for detecting GC and CRC. In an upper gastrointestinal endoscopy study of 60 pre-dialysis patients with CKD, Usta et al. observed significantly increased rates of erythematous gastritis, active chronic gastritis, and related findings (Usta et al., 2025). These conditions can progress to GC precancerous lesions and ultimately to cancer. Despite the high prevalence of gastrointestinal mucosal abnormalities in CKD patients, there are currently no CKD-specific guidelines for gastroscopic screening. Nevertheless, there are currently no CKD-specific guidelines for gastroscopic screening. In high-risk regions (e.g., Japan and South Korea), biennial endoscopic screening is recommended for adults older than 40 years (Cho and Jin, 2020). By contrast, in low-risk regions, screening may be discouraged or reserved for individuals with specific indications (e.g., hematemesis, hematochezia, or *H. pylori* infection). This variability underscores the absence of evidence-based recommendations tailored to CKD populations, and highlights the urgent need for prospective studies to determine optimal screening intervals and initiation thresholds in this high-risk group.

With respect to CRC, evidence indicates that CKD is associated with a significantly higher prevalence of polyps and adenomatous polyps compared with healthy controls (Madi et al., 2023). Because such lesions are established precursors to CRC, they may directly contribute to carcinogenesis. Currently, colonoscopy intervals for patients with CKD generally follow recommendations for the general population (e.g., the US Preventive Services Task Force), with repeat screening every 10 years after a normal colonoscopy (Gupta, 2022). However, it remains unclear whether this interval is appropriate for CKD patients, who may have accelerated carcinogenesis due to chronic inflammation, immune dysfunction, and uremic toxin exposure. Bowel preparation for colonoscopy is essential to ensure adequate mucosal visualization. However, this process is particularly challenging for dialysis patients, for whom strict fluid management is critical to avoid complications such as heart failure (Canaud et al., 2022). For patients who decline or are ineligible for colonoscopy, alternative modalities (computed tomographic colonography [CTC] and colon capsule endoscopy [CC]) substantially mitigate risks associated with high-volume fluid ingestion (Shaukat et al., 2021). Notably, positive findings on CTC or CC require confirmatory diagnostic colonoscopy (Shaukat et al., 2021). While these alternatives offer practical advantages in the dialysis population, their sensitivity and specificity for detecting premalignant lesions in CKD patients have not been rigorously evaluated. Emerging approaches, including artificial intelligence (AI)-assisted endoscopy, show promise for improving early detection of gastric and colorectal cancers, with reported accuracies of 80%–90% (Qu et al., 2020). Nonetheless, broader clinical adoption requires additional high-quality evidence.

To translate these findings into actionable clinical guidance, we propose the following considerations for CKD-specific screening strategies (Schell et al., 2022): Priority populations for screening: Patients with advanced CKD (stages 4–5), those on dialysis, and individuals with persistent proteinuria or rapidly declining eGFR should be considered high-risk and prioritized for gastrointestinal cancer screening. These patients may benefit from earlier and more frequent endoscopic evaluation (Huang et al., 2024). Selection of screening methods: For GC screening, gastroscopy remains the gold standard. In regions with high GC incidence, biennial gastroscopy is recommended for CKD patients over 40 years. For CRC screening, colonoscopy is preferred. However, in dialysis patients where fluid restriction is critical, non-invasive alternatives such as FIT may be considered as initial triage tools, though positive results should always be followed by colonoscopy. CTC and CC are acceptable alternatives when colonoscopy is not feasible (Danpanichkul et al., 2024). Surveillance intervals: Given the potential for accelerated carcinogenesis in CKD, a shorter colonoscopy interval (e.g., every 5 years) may be considered in high-risk subgroups, such as those with persistent proteinuria, inflammation, or prior adenomatous polyps. This approach, however, requires validation in prospective studies (Ahn et al., 2024). Special precautions for dialysis patients: Bowel preparation should be tailored to minimize fluid overload. Dialysis sessions should be scheduled close to the procedure to maintain fluid and electrolyte balance. In patients with significant comorbidities or intolerance to preparation, CTC or CC offer safer alternatives.

These recommendations are intended to bridge the gap between current evidence and clinical practice. However, they are not yet formal guidelines and highlight the urgent need for prospective trials to establish CKD-specific screening protocols.

In summary, while the elevated risk of DTT in CKD patients supports the rationale for enhanced screening, current recommendations are largely extrapolated from the general population. It must be clearly stated that there are no established CKD-specific screening guidelines, and well-designed prospective studies are urgently needed to generate the evidence base required for future clinical recommendations. Appropriately tailored screening strategies can optimize CKD management, particularly in dialysis settings, and improve clinical outcomes.

### 4.5 Balancing treatment benefits and risks

Throughout this review, we have presented evidence linking dialysis and kidney transplantation to an increased risk of digestive tract tumors. However, in clinical practice, these modalities are life-saving interventions for patients with end-stage renal disease (ESRD), and their benefits far outweigh the associated oncological risks for the majority of patients. A balanced perspective is therefore essential: rather than advocating avoidance of these treatments, we emphasize the importance of understanding and mitigating modifiable risk factors within the context of necessary renal replacement therapy.

#### 4.5.1 Dialysis adequacy and its dual role

Dialysis is indispensable for uremic toxin clearance and fluid balance, yet as discussed in Section 3.3, it may also promote chronic inflammation through blood-membrane interactions and dialysate impurities. This creates a paradox: inadequate dialysis exacerbates uremic toxin accumulation (Section 3.2), which directly promotes carcinogenesis; conversely, the dialysis procedure itself may contribute to inflammatory burden. Optimizing dialysis adequacy, that is, achieving sufficient small and middle molecule clearance while minimizing bioincompatibility, represents a potential strategy to reduce net oncogenic risk. Emerging evidence suggests that high-flux membranes and ultrapure dialysate may attenuate the inflammatory response (Zhou Z. et al., 2023; Ronco et al., 2025). Prospective studies are needed to determine whether enhanced dialysis biocompatibility translates into reduced long-term cancer incidence.

#### 4.5.2 Inflammation control as a therapeutic target

As detailed in Section 3.1, CKD is characterized by persistent systemic inflammation, which is further amplified by dialysis and contributes to both CKD progression and carcinogenesis. Targeting inflammation may therefore confer dual benefits: slowing renal deterioration and reducing cancer risk. Angiotensin-converting enzyme inhibitors (ACEi) and angiotensin receptor blockers (ARBs), already mainstays of CKD management, have been shown to attenuate NLRP3 inflammasome activation and reduce IL-1β production (Lin et al., 2022; Cortinovis et al., 2025). Whether these anti-inflammatory effects translate into reduced DTT incidence in CKD patients remains to be investigated. Similarly, emerging agents targeting the IL-1β pathway (e.g., canakinumab) have shown promise in reducing lung cancer incidence in population with cardiovascular disease (Garon et al., 2024); their potential role in CKD-associated malignancies warrants exploration.

#### 4.5.3 Infection prevention and management

Infection is a major complication of both dialysis (catheter-related bloodstream infections, peritonitis in PD) and transplantation (opportunistic infections under immunosuppression). Chronic or recurrent infections perpetuate systemic inflammation, indirectly promoting carcinogenesis through mechanisms described in Section 3.1. Moreover, certain infections have direct oncogenic potential. For example, EBV infection in transplant recipients is linked to lymphoepithelioma-like gastric carcinoma (Hirabayashi et al., 2023). Rigorous infection prevention strategies, including vaccination (e.g., hepatitis B, human papillomavirus), prophylactic antimicrobials where indicated, and prompt treatment of established infections, may therefore contribute to reducing infection-driven inflammation and, potentially, infection-associated malignancies.

#### 4.5.4 Immunosuppressive regimens: Balancing rejection prevention and cancer risk

Kidney transplantation is the optimal treatment for most patients with ESRD, offering superior survival and quality of life compared to long-term dialysis. However, as discussed in Section 3.3, immunosuppressive therapy is associated with a 2- to 3-fold increased risk of *de novo* malignancies, including GC and CRC. The key challenge lies in achieving sufficient immunosuppression to prevent allograft rejection while minimizing long-term cancer risk.

Current evidence suggests that cumulative immunosuppressive exposure is more important than any single agent in determining cancer risk (Vallianou et al., 2025; Sprangers et al., 2018). Nevertheless, differential effects among drug classes have been proposed: calcineurin inhibitors (CNIs; tacrolimus, cyclosporine) are associated with DNA damage and genetic instability in long-term users (Lizotti Cilião et al., 2016; Bedir et al., 2024), whereas mTOR inhibitors (sirolimus) have been hypothesized to exert antiproliferative effects and may be associated with lower cancer incidence (Nicolás-Morala et al., 2024). However, definitive evidence from randomized controlled trials is lacking, and mTOR inhibitors carry their own distinct side effect profiles that must be considered in clinical decision-making.

In clinical practice, individualizing immunosuppressive regimens, that is, considering each patient’s baseline cancer risk (e.g., age, prior malignancies, viral status), rejection risk, and comorbidities, represents a rational approach. For patients at particularly high cancer risk (e.g., those with pre-transplant malignancies or strong family history of GC/CRC), discussion of mTOR inhibitor-based regimens or minimization of CNI exposure may be warranted, while carefully balancing rejection risk.

### 4.6 Risk prediction and monitoring indicators

Throughout this review, several clinical parameters and biomarkers have emerged as potentially useful tools for risk stratification, screening initiation, and longitudinal monitoring of digestive tract tumors in patients with CKD. Synthesizing these indicators and understanding their current evidence base is essential for translating mechanistic insights into practical clinical applications and informing future research design.

#### 4.6.1 Renal function parameters

Proteinuria: As discussed in Sections 2 and 4.4, persistent proteinuria and its severity have been independently associated with increased CRC risk. Proteinuria is a readily available, low-cost clinical marker that could inform risk stratification and trigger earlier CRC screening. However, its utility as a standalone predictor is limited by the observational nature of the evidence and the lack of prospective validation. Future studies should examine whether proteinuria-guided screening improves clinical outcomes.

eGFR: The relationship between eGFR and DTT risk remains inconclusive. While some studies demonstrate increased DTT risk in pre-dialysis CKD patients, a Mendelian randomization analysis found no significant association between eGFR decrements and GC/CRC risk after adjustment for confounders (Tang et al., 2022). eGFR alone appears insufficient for risk prediction, but when combined with proteinuria and other markers, it may contribute to a composite risk score. The absence of standardized eGFR thresholds for screening initiation underscores the need for prospective cohort studies with serial eGFR measurements and detailed phenotyping.

#### 4.6.2 Inflammatory and metabolic biomarkers

Inflammatory markers: Section 3.1 detailed the central role of chronic inflammation in CKD-associated carcinogenesis, with IL-1β, IL-6, and TNF-α identified as key mediators. While these cytokines are plausible biomarkers for monitoring inflammatory burden and potentially predicting cancer risk, their clinical utility is limited by intra-individual variability, lack of standardized cutoffs, and absence of prospective data linking specific levels to DTT outcomes. Research priorities include validating inflammatory panels in CKD cohorts and testing whether anti-inflammatory interventions reduce cancer incidence.

Uremic toxins: Indoxyl sulfate and p-cresyl sulfate, discussed in Section 3.2, have been shown to promote CRC cell proliferation via AhR/c-Myc signaling. These toxins represent CKD-specific biomarkers that could theoretically stratify risk and guide timing of screening. However, measurement is not yet routine in clinical practice, and prospective studies are needed to establish threshold values associated with elevated cancer risk and to determine whether toxin-lowering interventions (e.g., AST-120, optimized dialysis) reduce DTT incidence.

VD and mineral metabolism indicators: VD deficiency, elevated FGF23, and disordered PTH, as discussed in Section 3.2, have been implicated in gastrointestinal carcinogenesis. VD status is easily measurable and modifiable, making it an attractive candidate for risk assessment. However, evidence linking these markers to DTT risk in CKD remains largely mechanistic or cross-sectional; longitudinal studies are required to establish their predictive value and to test whether correction of deficiencies alters cancer outcomes.

#### 4.6.3 Gut microbiota and metabolomic markers

Section 3.2 highlighted the role of gut dysbiosis in CKD, characterized by reduced SCFA-producing bacteria and expansion of uremic toxin-generating strains. Microbiota composition and fecal metabolites (e.g., SCFAs, p-cresol) hold promise as non-invasive biomarkers for risk stratification. However, the field faces significant challenges: inter-individual variability, lack of standardized analytical methods, and the absence of prospective studies linking specific microbial signatures to DTT development in CKD. Advances in metabolomics and large-scale cohort integration are needed before these markers can enter clinical use.

#### 4.6.4 Fecal immunochemical test (FIT)

As noted in Section 4.4, FIT has been used in CKD populations, with one prospective study detecting advanced colorectal neoplasia in 6.9% of CKD stages 3–5 patients. FIT is non-invasive, inexpensive, and widely available, making it an attractive option for CRC screening in CKD. However, its sensitivity and specificity specifically in CKD patients have not been rigorously evaluated, and positive predictive value may differ due to higher rates of non-neoplastic bleeding (e.g., from uremic gastropathy or angiodysplasia). Until CKD-specific validation is available, FIT should be interpreted cautiously, with a low threshold for colonoscopy following positive results.

## 5 CKD stratified risk framework for digestive tract tumors

To translate the dispersed evidence presented in this review into a clinically applicable framework, we herein propose a CKD-stratified risk framework for DTT. This framework integrates key renal parameters, including eGFR, proteinuria, dialysis modality, dialysis vintage, and kidney transplantation status, to stratify the risk of GC and CRC in patients with CKD. Table 2 summarizes the available evidence linking each stratification factor to DTT risk, with an indication of the strength and consistency of the supporting data based on the evidence grading discussed in Section 3.4.

**TABLE 2:** CKD-Stratified risk framework for digestive tract tumors (GC/CRC).

Risk stratification factor	Categories	Impact on DTT risk (GC/CRC)	Key evidence/References	Evidence strength
CKD Stage/Renal Function	General population	Reference	–	–
​	Low eGFR (as continuous variable)	No significant association with overall cancer or specific cancers (GC/CRC) after adjustment	Miyamoto et al. (2023) – prospective study; limitations: no proteinuria data, eGFR based solely on serum creatinine	Weak (null finding with methodological limitations)
​	Pre-dialysis CKD (unspecified stages)	Significantly increased risk of DTT (SIR 1.54), GC (SIR 1.60), and CRC (SIR 1.25)	Oh et al. (2018) – large Korean cohort (N = 35,443)	Moderate (single large cohort)
​	CKD stages 3–5	Advanced colorectal neoplasia detected in 6.9% of patients undergoing FIT-based screening	Au et al. (2022) – prospective cohort	Moderate
Proteinuria	None	Reference	–	–
​	Persistent proteinuria	Increased risk of CRC (aHR 1.27)	Oh et al. (2025)	Moderate (single large cohort)
​	Severity of proteinuria	Positive correlation with CRC risk	Oh et al. (2025)	Moderate
Dialysis Status	No dialysis (CKD non-dialysis)	Increased baseline risk (see CKD stage)	–	–
​	Hemodialysis (HD)	Increased risk of GC and CRC; diagnosed at earlier stages but higher mortality	Kida et al. (2022), Lee et al. (2018), Myung et al. (2020)	Strong (multiple cohorts)
​	Peritoneal dialysis (PD)	Higher GC risk compared to HD (∼2-fold); CRC risk also elevated	Lee et al. (2018): ∼2× GC risk; Kwon et al. (2019): HR 1.91 for overall malignancy	Moderate (confounding by indication)
Dialysis Vintage	Shorter duration	Lower cumulative risk (inferred)	–	Insufficient direct evidence
​	Longer duration	Higher cumulative risk likely due to prolonged uremic toxin exposure and inflammation	Consistent with mechanistic understanding	Inferred/Indirect
Kidney Transplantation	Pre-transplant (ESRD)	Elevated baseline risk from CKD/dialysis	–	–
​	Post-transplant (on immunosuppression)	Markedly increased risk of GC and CRC	Park et al. (2019), Apel et al. (2013) , Krynitz et al. (2013): SIR 1.8 (GC), 2.3 (CRC)	Strong (multiple cohorts)
​	Immunosuppressive regimen	Cumulative exposure more important than specific agent; potential differences (e.g., mTOR inhibitors) require further study	Vallianou et al. (2025), Sprangers et al. (2018) , Lizotti Cilião et al. (2016)	Moderate (mechanistic support)
Combined Factors	PD + long vintage + post-transplant	Highest theoretical risk	Integration of above	Hypothetical/Needs study

Abbreviations: CKD, chronic kidney disease; DTT, digestive tract tumor; GC, gastric cancer; CRC, colorectal cancer; eGFR, estimated glomerular filtration rate; SIR, standardized incidence ratio; aHR, adjusted hazard ratio; HD, hemodialysis; PD, peritoneal dialysis; ESRD, end-stage renal disease; mTOR, mammalian target of rapamycin; FIT, fecal immunochemical test.

This stratification framework is intended to: (i) Assist clinicians in identifying CKD patients at particularly elevated risk for GC and CRC. (ii) Guide the intensity and modality of cancer screening in this population. (iii) Highlight knowledge gaps where further research is needed to refine risk prediction. It must be emphasized that the current evidence remains largely observational, and causal relationships have not been definitively established. Therefore, this framework should be interpreted as a synthesis of available associations rather than a validated clinical prediction tool.

## 6 Conclusion and future directions

A growing body of observational and experimental evidence supports a close association between chronic kidney disease and digestive tract tumors, particularly gastric and colorectal cancers. Rather than serving merely as a background comorbidity, CKD establishes a systemic pro-tumorigenic milieu characterized by chronic inflammation, oxidative stress, metabolic reprogramming, immune dysregulation, and gut microbiota imbalance. These interconnected processes jointly impair gastrointestinal barrier integrity, weaken antitumor immune surveillance, and facilitate malignant initiation and progression.

Despite these advances, several critical gaps remain. First, causality has not been definitively established, and it remains unclear whether CKD independently drives gastrointestinal carcinogenesis or whether shared risk factors and bidirectional interactions contribute to disease progression. Large-scale, long-term prospective cohort studies incorporating renal function indices, proteinuria dynamics, inflammatory and metabolic biomarkers, and microbiome profiles are needed to clarify temporal and causal relationships. Second, most mechanistic insights are derived from experimental models, underscoring the need for translational studies that validate these pathways in human CKD populations.

From a clinical perspective, the absence of CKD-specific screening guidelines for gastric and colorectal cancers represents an unmet need. Risk-adapted screening strategies that integrate renal stage, dialysis modality, transplantation status, and metabolic disturbances may improve early detection and clinical outcomes. Emerging technologies, including artificial intelligence–assisted endoscopy and noninvasive biomarker-based screening tools, warrant further evaluation in CKD populations.

Future research should also prioritize the identification of modifiable targets, such as uremic toxins, gut microbial dysbiosis, to enable preventive and therapeutic interventions. Ultimately, a multidisciplinary approach that bridges nephrology, oncology, gastroenterology, and microbiome science will be essential for developing personalized strategies to reduce gastrointestinal cancer burden in patients with CKD.
